# Evaluation of the Tolerability and Efficacy of Adjuvant Treatment with Adipose Mesenchymal Stem/Stromal Cells in Dogs with Kidney Disease Secondary to Leishmaniosis

**DOI:** 10.3390/ani16213483

**Published:** 2026-11-04

**Authors:** Mario Alberto González, Rafael Barrera-Chacón, Joaquín Jiménez-Fragoso, Beatriz Macías-García, Javier Fernández-Cotrina, María Gil-Molino, Nicolás Roberto Robles, Francisco Eduardo Martín-Cano, Francisco Javier Duque

**Affiliations:** 1 Departamento de Medicina Animal, Hospital Clínico Veterinario, Universidad de Extremadura, 10003 Cáceres, Spain; marioalbgs@unex.es (M.A.G.); rabacha@unex.es (R.B.-C.); jojifra@unex.es (J.J.-F.); bemaciasg@unex.es (B.M.-G.); 2 Laboratorio LeishmanCeres (GLP Compliance Certified), Unidad de Parasitología, Hospital Clínico Veterinario, Universidad de Extremadura, 10003 Cáceres, Spain; javfercot@gmail.com; 3 Departamento de Sanidad Animal, Hospital Clínico Veterinario, Universidad de Extremadura, 10003 Cáceres, Spain; mariamilgm@unex.es; 4 Servicio de Nefrología, Hospital Universitario de Badajoz, Universidad de Extremadura, 06080 Badajoz, Spain; nrrobles@unex.es; 5 Laboratorio de Reproducción Equina y Espermatología, Hospital Clínico Veterinario, Universidad de Extremadura, 10003 Cáceres, Spain; femartincano@unex.es

**Keywords:** canine leishmaniosis, biomarker, scintigraphy, adipose mesenchymal stem cells

## Abstract

The tolerability and efficacy of treatment with allogeneic adipose-derived mesenchymal stem cells (aAMSCs) were evaluated in dogs with kidney disease secondary to canine leishmaniosis (CanL). In this non-blinded, single-center, 3-month study, twenty dogs received either conventional treatment (CT; *n* = 10) or conventional treatment plus an intravenous dose of aAMSCs (CT + aAMSCs; *n* = 10). Efficacy was assessed at days 0, 30, and 90 using plasma and urinary biomarkers. In both groups (CT, *n* = 6; CT + aAMSCs, *n* = 7), glomerular filtration rate was estimated by scintigraphy, and urinary proteomics was performed. No serious adverse events were observed, although two dogs experienced transient gastrointestinal events. No significant between-group differences occurred in renal parameters at time points. Intragroup analyses showed significant reductions in uNGAL/c in both groups and UPCR only in CT + aAMSCs. Longitudinal mixed-effects analyses revealed no significant group × time interactions for any of the evaluated variables. Urinary proteomics identified four protein entries with higher abundance in the CT + aAMSCs group at T90, although UMAP showed no separation between groups. A single intravenous aAMSCs dose was well tolerated but provided no renal benefits over conventional treatment. Further studies should evaluate alternative dosing protocols.

## Simple Summary

Kidney disease is an important complication of canine leishmaniosis that can negatively affect the health and survival of affected dogs. This study investigated whether adding a single intravenous dose of stem cells obtained from the fat tissue of healthy donor dogs to conventional treatment was well tolerated and could improve kidney health. Twenty dogs with naturally occurring kidney disease associated with leishmaniosis were followed for three months: ten received conventional treatment alone and ten received conventional treatment plus stem cells. The stem cell treatment was well tolerated, with no serious treatment-related adverse effects. Although some indicators of kidney damage changed over time in dogs receiving stem cells, no significant differences were found between the two groups, and an additional improvement in kidney function could not be demonstrated. These findings support the short-term tolerability of a single intravenous dose but do not demonstrate an additional clinical benefit. Future research resources should be directed toward evaluating whether repeated-dose protocols could provide greater benefits.

## 1. Introduction

In canine leishmaniosis (CanL), an inadequate host immune response plays a fundamental role in kidney pathology [1,2,3]. Among the complex network of pathogenic mechanisms involved, the deposition of immune complexes [2,4], and the inability of the macrophage to eliminate the parasite stand out [3,4]. These mechanisms underlie a hypersensitivity response, similar to other conditions such as human lupus nephritis (LN) [5,6] or other secondary glomerulonephritis in dogs [7,8].

Several treatment protocols are available; however, none has been demonstrated to be curative [2]. These treatments primarily aim to control the parasite and do not produce immediate effects on either the etiological agent or the host’s uncontrolled immune response. Consequently, kidney disease progresses in some individuals despite treatment [2,4]. Therefore, the search for complementary therapies is essential. One promising candidate is mesenchymal stem/stromal cells (MSCs) therapy, particularly adipose-derived (AMSCs). AMSCs have demonstrated effectiveness in various kidney pathologies, such as acute kidney injury induced by reperfusion/ischemia in canine [9,10], and murine models [11]. These studies reported less inflammation and cell proliferation in treated groups compared to untreated ones, leading to reduced parenchymal fibrosis and slower progression of kidney damage. MSCs therapy has also shown efficacy in chronic kidney conditions like diabetic nephropathy (DN) [12], IgA nephropathy (IgAN) [13] or LN [5,14] in murine models. The latter is particularly relevant because its pathogenic mechanisms are similar to those of CanL. In these models, MSCs therapy reduced humoral responses and lymph node activation, while promoting regulatory lymphocytic responses and immunotolerance [5,14]. Given these findings, AMSCs are being explored for treating diseases where the immune system plays a significant role, like glomerulonephritis secondary to CanL.

To evaluate kidney dysfunction and the response to treatment in CanL, various biomarkers are currently used [15]. However, their specificity and timing are limited, requiring the use of supplementary techniques. Scintigraphy is considered a precise method comparable to inulin clearance, the gold standard for evaluating glomerular function, offering greater speed and practicality [16]. Furthermore, mass spectrometry techniques are gaining relevance for accurately characterizing and comparing the urinary proteome on a large scale [17]. These techniques have already been applied in healthy dogs [18], and CanL-associated nephropathy [19], to monitor the response to conventional treatment [20], and in conditions with similar underlying pathogenesis, such as membranous glomerulonephritis associated with heartworms [8] and chronic kidney disease (CKD) in dogs [21,22].

Considering these factors, our study aimed to assess the tolerability and efficacy of adjuvant therapy using allogeneic AMSCs in dogs with kidney disease secondary to CanL, compared to conventional therapy. We hypothesized that AMSCs therapy would be well-tolerated and would result in improved kidney function compared to conventional treatment alone. To evaluate the tolerability, we monitored clinical signs and imaging findings during treatment and for 90 days post-treatment. To evaluate efficacy, we assessed kidney function parameters over 90 days and analyzed changes in the urinary proteome before and after treatment.

## 2. Materials and Methods

### 2.1. Study Design

This was a pilot, prospective, non-blinded, controlled, block-randomized study, conducted at a single center, with a follow-up period of 3 months. In this investigation, all procedures were reviewed by the Ethics Committee of the Office of the Environment and Rural Affairs, Agrarian Policies and Land, and Animal Health in Extremadura (Spain), No 2018208250016063. All dog owners were fully informed about the study procedures and provided written informed consent prior to their dogs’ enrollment in the study.

A total of twenty client-owned dogs were prospectively recruited from 2019 to 2022 at the Veterinary Teaching Hospital of Extremadura University. All dogs were diagnosed with advanced stages of CanL, specifically III and IV stages, according to the LeishVet classification criteria [23]. All of them were also diagnosed with CKD secondary to CanL, including only patients with renal ultrasound alterations indicating chronicity and proteinuria (urinary protein-creatinine ratio or UPCR ˃ 1), along with analytical and clinical changes consistent with CKD that persisted throughout the study, which are specified in said stages of the CanL [23]. Enrolled dogs were also classified according to the IRIS staging guidelines to assess CKD [24].

In the study, dogs were allocated to two treatment groups using permuted block randomization with a block size of two. Consecutively enrolled dogs were grouped into pairs, and a coin toss was used to randomly determine the treatment assignment of the first dog in each pair, while the second dog was allocated to the alternative treatment group. This procedure ensured balanced allocation between groups, with ten dogs assigned to each group. The conventional treatment group (CT) was treated with allopurinol (10 mg/kg twice daily, throughout the 90-day follow-up period) and miltefosine (2 mg/kg once daily for 28 days), and a second group received conventional treatment plus a single dose of allogeneic adipose-derived canine mesenchymal stem/stromal cells (aAMSCs) or CT + aAMSCs (Figure 1). Dogs in both groups were also treated with ramipril (0.125 mg/kg SID) as an antiproteinuric and antihypertensive agent. Oral phosphorus binders (calcium carbonate and chitosan, Ipakitine^®,^ Vetoquinol S.A., Lure, France) were also administered to dogs with hyperphosphatemia. No other therapeutic measures were used.

### 2.2. Patient Follow-Up

Dogs were evaluated at baseline, when they were diagnosed, and the corresponding treatment began to be administered (T0), at 30 days (T30) and at 90 days (T90) (Figure 1). At each visit, a thorough general physical examination was performed, including body weight and non-invasive systolic blood pressure measurement using Doppler ultrasonography (Vet BP Doppler^®^; Mano Médical, Lanvallay, France), along with a complete blood count and biochemical panel. Plasma concentrations of creatinine (pCr), urea (pUrea), cystatin C (pCysC), and symmetrical dimethylarginine (pSDMA) were evaluated as kidney function variables. Markers of kidney damage such as UPCR, urinary neutrophil gelatinase-associated lipocalin (uNGAL), and Tamm–Horsfall protein (uTHP) were analyzed in urine, and the urinary proteome was also studied using UHPLC-MS/MS. In addition, urinalysis and urine culture completed the kidney assessment and ruled out urinary infection.

Finally, echocardiography, complete abdominal ultrasound, and renal scintigraphy were performed at T0, T30, and T90, while chest radiographs were only performed at T0 and T90. These imaging tests were conducted at baseline and during follow-up to exclude pre-existing cardiopulmonary disease and to monitor potential related events associated with tolerability of intravenous aAMSCs administration.

### 2.3. Diagnosis of Canine Leishmaniosis

First, dogs with kidney disease and suspected CanL underwent a rapid immunochromatographic test (Uranotest Leishmania, Urano^®^vet Inc., Les Franqueses del Vallès, Spain; sensitivity 96.7%, and specificity 98.8%), which yielded positive results. In addition, specific antibodies against the total soluble antigen (SLA) of Leishmania infantum were detected using a semi-quantitative ELISA technique to confirm the diagnosis [25]. Moreover, the 4DX SNAP^®^ test (IDEXX Laboratories, Westbrook, ME, USA) ruled out other concomitant infectious diseases.

### 2.4. Canine Allogeneic Stem/Stromal Cell Therapy of Adipose Origin (aAMSCs)

#### 2.4.1. Isolation and Culture of aAMSCs

The vials containing the aAMSCs doses were supplied by Centauri Biotech SL (Arteixo, Spain). The processing protocol for cell therapy involved the extraction of subcutaneous fat samples from the gluteal region of five young male dogs, as described in the literature [26]. Five grams of fat were digested with 0.1% collagenase type II. After digestion, the primary cultures were separated and expanded in Dulbecco’s Modified Eagle Medium (DMEM). All reagents were sourced from Sigma-Aldrich (Merck KGaA, Darmstadt, Germany). Once the cultures reached 80% confluency, the cells were harvested using 0.25% trypsin. Subculturing continued until passage 2. Subsequently, the cells were harvested again and concentrated to 10^6^ cells/mL in a cryopreservation solution (10% dimethyl sulfoxide, 90% fetal bovine serum or FBS). The cells were then aliquoted into 1 mL vials at −80∘C, following an established protocol [27,28,29].

#### 2.4.2. Characterization and Multipotentiality of aAMSCs

Adipose tissue samples were obtained from healthy donor dogs. Cells from each donor were isolated, cultured, expanded, and cryopreserved separately. Each administered cell dose was derived from a single donor, without pooling cells from different donors. To characterize aAMSCs at passage 2, fluorescence-activated cell sorting or FACS was employed [27,28]. The cells showed positive expression of mesenchymal markers (CD29, CD90, and STRO-1) and negative expression of hematopoietic markers (CD34 and CD45) and major histocompatibility complex class II (MHC-II) [27]. Multipotency was assessed using established protocols, evaluating the differentiation capacity of aAMSCs into adipogenic, chondrogenic, and osteogenic lineages [27,30]. Cell viability was assessed before the beginning of the study and reassessed following its completion, with values consistently ranging from 70% to 80%, indicating that cell viability was maintained throughout the study period, in accordance with previously published findings [29].

#### 2.4.3. Administration and Tolerability Evaluation of Stem/Stromal Cell Therapy

The cryopreserved vials were thawed in a thermostatic bath set between 30 and 35 °C and resuspended in 100 mL of prewarmed 0.9% saline at the same temperature. At T0, dogs in CT + aAMSCs group received 1–1.5 × 10^6^ cells per kilogram via the cephalic vein over 45 min, and then the dogs continue with the conventional treatment. During infusion, heart rate, respiratory rate and pattern, rectal temperature, and noninvasive Doppler blood pressure were monitored every 5 min. Dogs were observed for at least 6 h after administration, exceeding the observation period previously described [28,29]. Tolerability was further assessed through clinical examinations and imaging studies (echocardiography, complete abdominal ultrasound, and chest radiographs) until day 90.

### 2.5. Evaluation of Kidney Function

#### 2.5.1. Blood Biomarkers

Heparinized plasma samples from the jugular vein were utilized for analysis. pCr and pUrea were determined using colorimetric techniques: creatinine by the Jaffe method (gernon, RAL^®^, Barcelona, Spain) and urea by the urease-glutamate dehydrogenase method (Spinreact^®^, Barcelona, Spain). pCysC was measured using a human latex turbidimetric assay validated for canine species (Cystatin C turbilatex; Spinreact^®^, Barcelona, Spain) [31]. All analyses were conducted using automated equipment (Saturno 100; CRONY^®^ instruments; Rome, Italy). Finally, pSDMA was measured using the IDEXX SDMA^®^ Test (IDEXX Laboratories; Westbrook, ME, USA).

#### 2.5.2. Urinary Biomarkers

Urine samples collected via ultrasound-guided cystocentesis were immediately centrifuged. The urinary supernatant was used to measure the UPCR, while the remaining urine was frozen at −80 °C for future analysis. The UPCR was calculated using protein levels determined by a colorimetric technique (pyrogallol red, gernon, RAL^®^, Barcelona, Spain) and creatinine levels measured using the same method as for blood samples. Both were analyzed using the Saturno 100 automatic analyzer (CRONY^®^ instruments; Rome, Italy). Finally, quantitative sandwich ELISA techniques were employed to measure uNGAL (Canine NGAL ELISA kit; Abcam, Inc.; Cambridge, UK; No. ab205085) and uTHP (Canine T-H glycoprotein ELISA kit; MyBioSource, Inc.; San Diego, CA, USA; No. MBS010151). Results of uNGAL and uTHP were expressed as a ratio of urinary biomarker levels to creatinine, and samples were analyzed in duplicate.

#### 2.5.3. Renal Scintigraphy and eGFR

As described in the literature [32,33,34,35,36], doses ranging from 37 to 148 MBq of 99m-labeled technetium diethylenetriaminepentaacetic acid (99mTc-DTPA) were administered via the cephalic vein to estimate the glomerular filtration rate (eGFR) with the dog positioned in left lateral recumbency. A dynamic study was conducted using a dorsal view with an Equine Scanner H.R system equipped with a Scitron VI gamma camera. Subsequently, a static study was performed from the right lateral abdominal view to calculate depth and attenuation using the modified Gate’s method [32,33,34].

#### 2.5.4. Statistical Analysis

Statistical analyses were performed using SigmaPlot version 12.0. Descriptive statistics were calculated, and normality was assessed using the Shapiro–Wilk test. At each time point, normally distributed variables were compared between groups using Student’s *t*-test, whereas non-normally distributed variables were compared using the Mann–Whitney rank-sum test. Within-group comparisons across time points were performed using repeated-measures one-way ANOVA for normally distributed variables and the Friedman test for non-normally distributed variables. When significant differences were detected, Holm–Šidák multiple comparisons were performed.

Additionally, linear mixed-effects models were applied to all variables regardless of the statistical significance of previous intragroup comparisons. All variables were log-transformed before analysis. Treatment group, time (T0, T30, and T90), and their interaction were included as fixed effects, with a random intercept for each dog to account for repeated measurements. Time was treated as a categorical variable. Models were fitted using maximum likelihood, and the overall group × time interaction was evaluated using likelihood-ratio tests comparing models with and without the interaction term. Analyses were performed using Python (version 3.13.5) and statsmodels (version 0.14.6). Additionally, baseline-adjusted mixed-effects models were performed using T30 and T90 measurements as dependent variables and the corresponding T0 value as a covariate. In all of them, statistical significance was defined as *p* < 0.05.

### 2.6. Proteomic Analysis

#### 2.6.1. Urinary Protein Processing and Mass Spectrometry Analysis

The required volume of urine supernatant was calculated based on protein concentration from UPCR. Samples were stored at −80 °C after centrifugation to 200 g for 5 min. Following thawing, urinary proteins were subjected to tryptic digestion according to a previously published protocol [19] using porcine Trypsin Gold (Mass Spectrometry Grade; Promega). Peptide separation and analysis were performed using an ultra-high-performance liquid chromatography system (UHPLC; Agilent 1290 Infinity II System, Agilent Technologies, Santa Clara, CA, USA) coupled to tandem mass spectrometry (MS/MS; Agilent 6550 Q-TOF, Agilent Technologies, Santa Clara, CA, USA) equipped with an Agilent Jet Stream Dual electrospray ionization source, operating in positive ion mode. Each urine sample was analyzed in duplicate by UHPLC-MS/MS, and the mean of the two technical replicates was used for subsequent bioinformatic analyses, with each dog considered an independent biological unit.

Data were acquired in data-dependent acquisition or DDA mode. Peptides were separated using an AdvanceBio Peptide Mapping column (2.7 μm, 150 × 2.1 mm; Agilent Technologies) at a flow rate of 0.4 mL/min and maintained at 55 °C. MS and MS/MS data were acquired over a mass range of 50–1700 *m*/*z*. Raw data were processed using Spectrum Mill MS Proteomics Workbench Software (Rev B.04.01; Agilent Technologies). Spectral extraction was performed using default parameters, including a precursor mass range of 50–10,000 *m*/*z*, maximum precursor charge state of +5, retention time tolerance of ±60 s, and a minimum signal-to-noise ratio of 25.

Database searching was performed against the UniProt *Canis lupus familiaris* protein database (downloaded in April 2023; https://www.uniprot.org), assuming trypsin specificity and allowing up to five missed cleavages. Carbamidomethylation of cysteine residues was considered a fixed modification, while methionine oxidation was included as a variable modification. Precursor and fragment ion mass tolerances were set at 20 ppm and 50 ppm, respectively. Peptide and protein identifications were validated using the Spectrum Mill autovalidation algorithm. An initial peptide-level false discovery rate (FDR) threshold of 1.2% was applied using an automated target-decoy strategy (auto-threshold mode). Subsequently, a protein-level polishing step was performed using a stringent target protein FDR of 0% to ensure high-confidence identifications. Only proteins identified with at least two unique peptides were retained for downstream statistical and bioinformatic analyses. Relative protein abundance was assessed using a label-free approach based on peptide ion intensities. This conservative filtering strategy was applied to minimize false-positive identifications, considering the exploratory nature of the study and the limited sample size. The entire analytical and data processing workflow was performed as previously described [19,37].

#### 2.6.2. Bioinformatics

Data normalization and logarithmic transformation were performed prior to statistical analysis. Low-variance features were excluded using variance filtering to reduce background noise and improve the robustness of downstream analyses [38]. All analyses were conducted using Qlucore Omics Explorer version 3.6 (Qlucore, Lund, Sweden; https://qlucore.com).

For unsupervised exploration of the dataset, Uniform Manifold Approximation and Projection (UMAP) was applied to visualize global urinary protein abundance patterns and assess potential separation between experimental groups. The UMAP representation was revised to display one point per biological unit. This approach avoided the visual overrepresentation of technical replicates, which had originally been plotted separately. Principal component analysis (PCA) was additionally performed within each experimental group to explore potential intragroup variability and sample dispersion in the proteomic dataset.

Differential protein abundance analysis was performed using Qlucore Omics Explorer version 3.6 by fitting a linear model for each protein variable to compare the experimental groups. Multiple testing correction was applied using the Benjamini–Hochberg false discovery rate (FDR) procedure [39]. Adjusted *p*-values (q-values) < 0.1 were considered statistically significant for this exploratory analysis [40], while a more restrictive threshold of q < 0.07 was applied for the final selection of differentially abundant protein entries, following the general framework of FDR-based multiple testing in high-dimensional biological studies [40]. This threshold was selected considering the exploratory nature of the study and its limited biological sample size. Fold-change, *p*-value, and q-value thresholds were used as statistical filtering criteria to identify potentially differentially abundant proteins. These criteria represented selection thresholds rather than identical individual statistical estimates for all selected protein entries. Individual Tukey-adjusted *p*-values obtained from subsequent comparisons were presented in the corresponding protein abundance plots.

Functional enrichment analysis of statistically significant proteins was performed using the g:Profiler web server (https://biit.cs.ut.ee/gprofiler/gost) (accessed on 25 April 2025). Only annotated genes were considered, excluding electronically inferred annotations, and the g: SCS multiple testing correction was applied with a significance threshold of *p* < 0.01.

## 3. Results

### 3.1. Animals

A total of 20 dogs of different breeds were enrolled in the study and randomly allocated to two treatment groups (*n* = 10 each). At baseline, the CT group included three females and seven males, with a mean age of 4.7 years, whereas the CT + aAMSCs group comprised six females and four males, with a mean age of 6.3 years. Regarding kidney function, median plasma creatinine concentrations were 1.35 mg/dL in the CT group and 1.95 mg/dL in the CT + aAMSCs group, while median UPCR values were 4.84 and 3.35, respectively. According to the IRIS classification, the CT group included two dogs in stage 1, six in stage 2, and two in stage 3. In contrast, the CT + aAMSCs group included three dogs in stage 1, four in stage 2, two in stage 3, and one in stage 4. These findings indicate some baseline imbalances between groups, particularly in age, sex distribution, plasma creatinine, UPCR, and disease severity. The complete individual baseline characteristics and longitudinal clinical and laboratory measurements are presented in Supplementary Table S1. According to the LeishVet classification, four dogs in the CT group were classified as stage III and six as stage IV, whereas six dogs in the CT + aAMSCs group were classified as stage III and four as stage IV.

All dogs classified as LeishVet stage III or IV in both treatment groups were proteinuric (UPCR ˃ 1). Three dogs in each group had hypertension, which remained controlled during the 3-month follow-up with antiproteinuric medication. No additional antihypertensive treatment was required. One dog in each group received phosphorus binders during the study.

During follow-up, three dogs died following progression of their pre-existing chronic kidney disease: two in the CT group (days 32 and 41) and one in the CT + aAMSCs group (day 35). The deceased dog in the CT + aAMSCs group (MSC-08) was classified as IRIS stage 4 and LeishVet stage IV at baseline, with plasma creatinine of 5.4 mg/dL and UPCR of 12.97. The two deceased dogs in the CT group (CT-02 and CT-04) were classified as IRIS stages 3 and 2, respectively, both being LeishVet stage IV, with baseline creatinine concentrations of 2.9 and 1.9 mg/dL and UPCR values of 3.38 and 6.43, respectively (Supplementary Table S1). Necropsies were performed on all three deceased dogs to investigate the causes of death. Based on the available clinical, laboratory, and post-mortem findings, the deaths were attributed to progression of the underlying kidney disease, with no evidence directly implicating aAMSCs administration. Consequently, the T90 assessments included eight surviving dogs in the CT group and nine in the CT + aAMSCs group.

Scintigraphy-derived eGFR and urinary proteomic analyses were performed in a subgroup of 13 dogs (CT, *n* = 6; CT + aAMSCs, *n* = 7). The reduced sample size for these complementary assessments was due to financial constraints and the refusal of some owners to provide consent for these additional procedures, which prevented their application to the entire study population.

### 3.2. Short-Term Tolerability to aAMSCs Injection

The tolerability of aAMSCs therapy was evaluated during treatment administration (T0) and at subsequent follow-up visits on days 30 (T30) and 90 (T90). During infusion, two of the ten dogs receiving aAMSCs (20%) experienced transient gastrointestinal adverse events: one developed nausea and the other vomited. These adverse effects were managed successfully by reducing the rate of aAMSCs infusion and administering a single dose of intravenous maropitant (1 mg/kg). No abnormalities attributable to aAMSCs administration were detected on follow-up echocardiography or abdominal ultrasonography at days 30 and 90, or on chest radiography at day 90.

### 3.3. Blood and Urinary Biomarkers

No significant between-group differences were detected in plasma creatinine, urea, cystatin C, or SDMA at any evaluated time point (all *p* > 0.05). Similarly, no significant intragroup differences were identified, although numerical changes were observed during follow-up. Furthermore, the rest of the blood parameters tested related to CKD, such as albumin, calcium, phosphorus and potassium, did not show differences among groups. However, only albumin showed a significant intragroup difference in the CT group between T0 and T90 (Table 1).

To further investigate these findings and determine whether the observed changes over time differed between treatment groups, longitudinal mixed-effects analyses were performed, allowing for a direct assessment of the group × time interaction. However, no statistically significant group × time interactions were observed for plasma creatinine (*p* = 0.745), urea (*p* = 0.554), cystatin C (*p* = 0.265), or SDMA (*p* = 0.621). Similarly, no significant interactions were identified for albumin (*p* = 0.241), calcium (*p* = 0.821), phosphorus (*p* = 0.931), or potassium (*p* = 0.317). Therefore, the longitudinal evolution of the evaluated plasma biochemical parameters did not differ significantly between treatment groups.

To account for potential baseline imbalances between treatment groups, additional longitudinal mixed-effects analyses were performed after adjustment for the corresponding baseline values. No statistically significant group × time interactions were identified for plasma creatinine (*p* = 0.763), urea (*p* = 0.743), cystatin C (*p* = 0.082), SDMA (*p* = 0.258), albumin (*p* = 0.115), calcium (*p* = 0.671), phosphorus (*p* = 0.823), or potassium (*p* = 0.233). These results were consistent with the unadjusted analyses, as no statistically significant differences in longitudinal trajectories were demonstrated between treatment groups.

Regarding urinary biomarkers, no statistically significant differences in uNGAL/c or uTHP/c were observed between treatment groups at any evaluated time point (Table 2). However, intragroup comparisons revealed a statistically significant reduction in uNGAL/c between T0 and T90 in both the CT and CT + aAMSCs groups, whereas no significant changes in uTHP/c were identified within either group. Additionally, longitudinal linear mixed-effects models were applied to the urinary biomarkers to assess whether changes over time differed between treatment groups. No statistically significant group × time interactions were identified for uNGAL/c (*p* = 0.666) or uTHP/c (*p* = 0.603). These findings indicate that the longitudinal evolution of the evaluated urinary biomarkers did not differ significantly between the CT and CT + aAMSCs groups, despite the significant intragroup changes observed for some parameters.

Finally, no statistically significant differences in UPCR were observed between treatment groups at any of the evaluated time points (Table 2). Intragroup analysis revealed a significant reduction in UPCR between T0 and T90 in the CT + aAMSCs group (*p* = 0.019), whereas the reduction observed in the CT group did not reach statistical significance (*p* = 0.113). Nevertheless, the longitudinal mixed-effects analysis showed no significant group × time interaction for UPCR (likelihood-ratio test, *p* = 0.859), indicating that the longitudinal evolution of proteinuria was not significantly different between the two treatment groups.

Similarly, baseline-adjusted longitudinal mixed-effects analyses revealed no statistically significant group × time interactions for uNGAL/c (*p* = 0.580), uTHP/c (*p* = 0.775), or UPCR (*p* = 0.570). Therefore, adjustment for baseline values did not change the overall interpretation of the urinary biomarker results.

### 3.4. Estimated Glomerular Filtration Rate by Scintigraphy

Scintigraphy-derived eGFR was assessed in 13 dogs (CT, *n* = 6; CT + aAMSCs, *n* = 7). The reduced sample size for scintigraphy-derived eGFR and urinary proteomic analyses was due to financial constraints and the refusal of some owners to consent to these additional procedures, which prevented their application to the entire study population. As shown in Figure 2, numerical increases in eGFR were observed during follow-up in both treatment groups. However, these changes did not reach statistical significance in either the CT group (*p* = 0.220) or the CT + aAMSCs group (*p* = 0.430). Similarly, no statistically significant differences were identified between groups at T0 (*p* = 0.910), T30 (*p* = 0.507), or T90 (*p* = 0.887). Longitudinal mixed-effects analysis revealed no statistically significant group × time interaction (*p* = 0.866), and this finding remained unchanged after adjustment for baseline eGFR (*p* = 0.743).

### 3.5. Urinary Proteomics

The urinary proteome of 6 dogs in the CT group and 7 dogs in the CT + aAMSCs group was analyzed and compared. A total of 368 proteins were identified. Initially, a baseline urinary proteomic comparison between the CT and CT + aAMSCs groups was performed at T0, before the administration of cell therapy, to assess potential pre-existing differences in protein abundance between treatment groups. As no statistically significant differences were identified at baseline, these results were not graphically represented, and the subsequent proteomic analyses and visualizations focused on the post-treatment time points (T30 and T90). UMAP was used to visualize the distribution of individual dogs based on their urinary proteomic profiles at T30 (Figure 3A) and T90 (Figure 3B). In these graphics, there was no clear separation of individuals from the two study groups. Moreover, PCA was performed as an unsupervised exploratory approach to assess intra-group variability. However, it did not reveal any clear separation or consistent clustering, indicating a high degree of overlap in proteomic profiles. Functional enrichment analysis was conducted using g:Profiler on the set of statistically significant proteins, applying multiple testing correction (g: SCS method, *p* < 0.01). No significantly enriched biological pathways or functional categories were identified. Accordingly, PCA and functional analyses are reported in the text but not presented as figures, as they did not contribute additional biological interpretation to the results.

Subsequently, differential urinary protein abundance between treatment groups was evaluated at each post-treatment time point. At T30, no statistically significant differences were identified between groups, although four protein entries showed a tendency towards higher abundance in the CT + aAMSCs group. At T90, these four protein entries met the filtering criteria applied in Qlucore Omics Explorer (FC ≥ 2.85, *p* ≤ 0.007, and q < 0.07), showing higher abundance in the CT + aAMSCs group. The descriptive FCs were 7.46 for osteopontin (E2R161), 7.12 for osteopontin (A0A5F4BRW4), 95,03 for WAP four-disulfide core domain protein 2 (F1PDT8), and 68.70 for peptidase S1 domain-containing protein (F1PCE8). Their respective Tukey-adjusted *p*-values were 0.0046, 0.0046, 0.00039, and 0.00061 (Figure 4). All four entries satisfied the q-value filter, although individual q-values were not extracted.

## 4. Discussion

In dogs, aAMSCs therapy has proven to be a well-tolerated and effective alternative for treating various diseases [28,29]. It has even been proposed as a potential candidate for the treatment of acute kidney injury (AKI) [10] and CKD [41]. Adipose-derived cells are also considered more practical because of the accessibility of adipose tissue and the relative ease of cell isolation and culture compared with other tissue sources. Regarding tolerability, AMSCs exhibit lower immunogenicity and greater secretory activity than MSCs from other sources, such as those derived from bone marrow [42,43]. Their low immunogenicity is attributed to the lack of type II major histocompatibility complex (MHC) and costimulatory molecules. Furthermore, these cells express low levels of MHC class I molecules compared with other cell types [42,44,45,46]. These immunological characteristics may facilitate allogeneic MSCs administration, although donor–recipient DLA compatibility was not assessed in the present study [46]. This therapy has also been demonstrated to be tolerated in feline kidney disease [47] and rodent models [13,14]. Consequently, numerous studies have investigated this therapeutic approach.

Although the administration protocol is not fully established, many studies report the intravenous administration of aAMSCs therapy without major adverse effects in dogs with inflammatory bowel disease [29], atopic dermatitis [28], and CKD [41]. In our case, only two individuals experienced nausea and vomiting, which subsided with a decreased infusion rate and administration of antiemetics. One possible explanation for these adverse events is the use of dimethyl sulfoxide (DMSO) as a cryoprotectant, as its infusion may induce nausea and vomiting [48,49]. Despite this, its intravascular use in dogs at a concentration of 10% is considered safe; nonetheless, its toxicity may be increased in dogs with CKD [48]. FBS could also explain these effects, although the most frequently reported effects include local reactions and the development of antibodies against bovine proteins [50,51]. Despite this, currently no alternative has been found that prevents the alteration of the characteristics, viability and potentiality of MSCs during storage [51]. Although our procedure followed protocols used in previous canine studies [29] and the supplier’s recommendations, washing the aAMSCs before administration might reduce these reactions. Therefore, washing and alternatives to cryoprotectants and preservatives such as DMSO and FBS, and preservative-free protocols are currently under study and should be considered in the future [49,51]. Additionally, monitoring with diagnostic imaging tests (abdominal ultrasound, echocardiography, and chest radiography) showed no additional alterations after 90 days. These findings are consistent with previous reports [41] and suggest that intravenous aAMSCs administration was generally well tolerated in dogs with CanL-associated kidney disease.

Regarding the efficacy of aAMSCs therapy, our goal was to leverage the primary role of MSCs’ immunomodulatory potential to alleviate the proinflammatory environment [42], which contributes to the progression of kidney damage until the parasite is controlled, and even after. Despite this, the results did not reveal statistically significant differences among groups, or in the intragroup comparison at the different time points evaluated, in any of the blood biomarkers used to assess kidney function and related glomerular filtration, namely, pCr, pUrea, pCysC, pSDMA and eGFR. In parallel, other blood parameters such as phosphorus, calcium or potassium did not vary. Albumin was the only parameter that showed significance within the CT group between times T0 and T90. However, both values remained below the lower reference limit of 2.5 g/dL, and the observed difference was not considered clinically relevant.

These findings are consistent with previous studies suggesting that their regenerative potential is not specifically directed at the glomerulus; instead, it has only been demonstrated at the tubular level with other MSC sources, such as intravenous bone marrow stem/stromal cells (bmMSCs) in a murine model of AKI [45,52]. This regenerative potential could be enhanced by aAMSCs, which have been demonstrated to alter the microenvironment when tubular damage occurs, facilitating transformation to an epithelial lineage in vitro [53]. In the present study, reductions in urinary tubular biomarkers and proteinuria were observed during follow-up in both treatment groups. Although significant within-group reductions in uNGAL/c were detected in both groups, and UPCR decreased significantly only in the CT + aAMSCs group, these findings should not be interpreted as evidence of an additional therapeutic effect attributable to aAMSCs administration. To address this issue, additional longitudinal mixed-effects analyses were performed to directly compare the temporal evolution of UPCR and uNGAL/c between treatment groups. Neither variable showed a statistically significant group × time interaction. Importantly, all dogs received ramipril as part of conventional treatment, which may have contributed to the observed reductions in proteinuria. Therefore, despite the observed reductions in urinary biomarkers, no additional effect of aAMSCs administration on their longitudinal trajectories could be demonstrated.

Previous experimental studies have described the immunomodulatory and potential renoprotective properties of mesenchymal stromal cells, including modulation of inflammatory pathways, reduction in transforming growth factor-β (TGF-β) signaling, and attenuation of kidney fibrosis [11,13,54]. Nevertheless, these mechanisms were not directly evaluated in the present study, and the observed changes in urinary biomarkers cannot be attributed specifically to aAMSCs therapy.

Moreover, no differences were observed in the urinary proteome between the CT and CT + aAMSCs groups after the first month of treatment; however, a trend towards increased abundance of four proteins was detected in the CT + aAMSCs group. At T90, four protein entries showed higher relative abundance in the CT + aAMSCs group and met the Qlucore filtering criteria, including two entries annotated as osteopontin, which may represent distinct protein forms or database annotations. Osteopontin, which has already been described in the urinary proteome of kidney disease secondary to CanL, was found to be elevated compared to healthy dogs [19]. Its increase is related to the inflammatory and fibrogenic pathogenesis of several diseases such as DN and LN [55]. WAP four-disulfide core domain protein 2 is a protease inhibitor identified as a profibrotic biomarker in human diabetic nephropathy [56]; however, its presence in canine urine has not previously been described. Lastly, peptidase S1 domain-containing protein has been noted in dogs with CanL [19], and its increase in urine is associated with lower survival [20]. It also shows high similarity to Cationic trypsin-like protein, which is related to acute pancreatitis. Although its presence in urine has not been directly linked to kidney disease pathogenesis, as a protease, it could be involved in the fibrosis process and EMT. Nevertheless, these previously reported associations do not establish a causal relationship between the increased urinary abundance of these proteins and aAMSCs administration. Although these proteins have been associated with pathological processes, their higher abundance in the treated group cannot be interpreted as causal evidence that cell therapy promoted kidney fibrosis or accelerated disease progression. This conclusion is further supported by the lack of significant separation among individuals from both groups in the bioinformatic study represented by UMAP.

Although no significant urinary proteomic differences were detected at T0, pre-existing histopathological differences between groups cannot be excluded, as these may not have been reflected in the urinary proteome initially but could have become apparent during disease progression. However, this hypothesis could not be confirmed because systematic kidney histopathological assessment was not performed for ethical and practical reasons. Although necropsies were conducted on the three dogs that died during follow-up, these examinations did not constitute a standardized histopathological evaluation of kidney lesions across treatment groups. This represents a limitation of the study. Nevertheless, these results warrant further investigation, including the evaluation of multiple-dose protocols, to clarify the potential role of aAMSCs therapy in kidney disease associated with CanL.

In our study, the MSCs were administered intravenously, as this route is more practical and accessible, and is not associated with serious adverse reactions. Although pulmonary sequestration has been observed in several studies and may affect treatment viability and efficacy [57,58], other studies have not reported this phenomenon. For example, Morigi et al. (2004) reported that intravenously administered bone marrow-derived mesenchymal stem/stromal cells (BM-MSCs) were detected within the tubular epithelium in a murine model of cisplatin-induced acute kidney injury, as demonstrated by Y-chromosome detection [52]. In another study involving a rat model of CKD, human AMSCs administered via the tail vein were detected in the kidney parenchyma using the cell marker CMFDA, with associated improvements in kidney function and fibrosis [59]. Moreover, an emerging hypothesis suggests that even without direct localization in the injured organ, aAMSCs can secrete substances from nearby locations in response to the injury site through paracrine and even endocrine mechanisms [11,42,44,60].

Regarding cell type, aAMSCs therapy is a promising choice for future research due to its advantages over other MSC sources, including bmMSCs [43,45,61]. Moreover, they have been shown to be effective in kidney disease models similar to CanL such as systemic lupus erythematosus, specifically in a murine model where intravenous aAMSCs led to a decrease in proinflammatory Th1 and Th17 cells and an increase in the immunotolerant Treg response, which was accompanied by reduced inflammatory cell infiltration and inflammation in the mesangium and interstitium [5].

Finally, with respect to the frequency of treatment, a recent study on CKD in dogs reported that the administration of three doses of aAMSCs improved creatinine levels compared to the control group, but differences were not significant; however, a clear clinical improvement was reported in the evaluated dogs [41]. Nonetheless, this study included patients with CKD whose pathophysiology differs from the secondary glomerulonephritis due to immune complexes described in kidney disease associated with CanL, in which immunopathogenic mechanisms are the primary cause of tissue damage. Our choice of a single dose administration of AMSCs was based on the lack of previous literature on the topic. Furthermore, this kind of protocol has shown promising results in rodent models of kidney fibrosis [45,59], and in pathologies that exhibit similarities to glomerulonephritis secondary to CanL, such as LN in both rodent studies [5] and advanced human cases [62]. Nevertheless, a single intravenous dose of aAMSCs did not produce significant improvements in kidney function parameters in the present study. Thus, although single-dose protocols have shown benefits in other conditions [28,29], a single dose may be insufficient to improve kidney function in dogs with CanL-associated CKD.

Several limitations should be considered when interpreting these findings. Despite random allocation, baseline imbalances were observed between treatment groups in age, sex distribution, kidney function biomarkers, proteinuria, and IRIS stage, which may have influenced treatment responses. To address this concern, additional longitudinal mixed-effects analyses adjusted for baseline values were performed. These analyses did not identify statistically significant group × time interactions for any of the evaluated variables, supporting the interpretation of the unadjusted findings. Nevertheless, the small sample size limits statistical power and the precision of treatment effect estimates. Furthermore, scintigraphy-derived eGFR and urinary proteomic analyses were restricted to 13 dogs because of financial constraints and the refusal of some owners to consent to these complementary procedures, potentially introducing selection bias. Three dogs also died during follow-up due to progression of their pre-existing kidney disease, resulting in missing T90 observations and potential attrition bias. Therefore, these findings should be interpreted cautiously, and larger studies with more balanced baseline characteristics and complete longitudinal follow-up are warranted.

In addition, the evaluation of a single intravenous dose and the relatively short follow-up period limit the conclusions that can be drawn regarding the potential efficacy of this therapeutic approach. Future studies should investigate alternative administration protocols, including repeated doses or different cell concentrations, to further evaluate the potential benefits of adjuvant aAMSCs therapy, as suggested by previous studies on kidney pathology in murine models [13,14]. Additionally, future research could explore the potential therapeutic role of mesenchymal stem/stromal cell-derived extracellular vesicles in kidney disease associated with CanL.

## 5. Conclusions

A single intravenous dose of aAMSCs was generally well tolerated, with no serious adverse events identified as directly attributable to cell administration during the study period. However, no additional improvement in kidney function was demonstrated compared with conventional treatment alone. Although reductions in proteinuria and urinary biomarkers of tubular injury were observed during follow-up, longitudinal mixed-effects analyses, including baseline-adjusted models, revealed no statistically significant group × time interactions for any of the evaluated kidney function or damage biomarkers, indicating that these changes could not be attributed specifically to aAMSCs administration. Given the exploratory nature of this pilot study, the limited sample size, and the use of a single-dose protocol, further controlled studies are needed to assess whether alternative administration regimens, particularly repeated doses, may provide additional renal benefits.

## Figures and Tables

**Figure 1:**
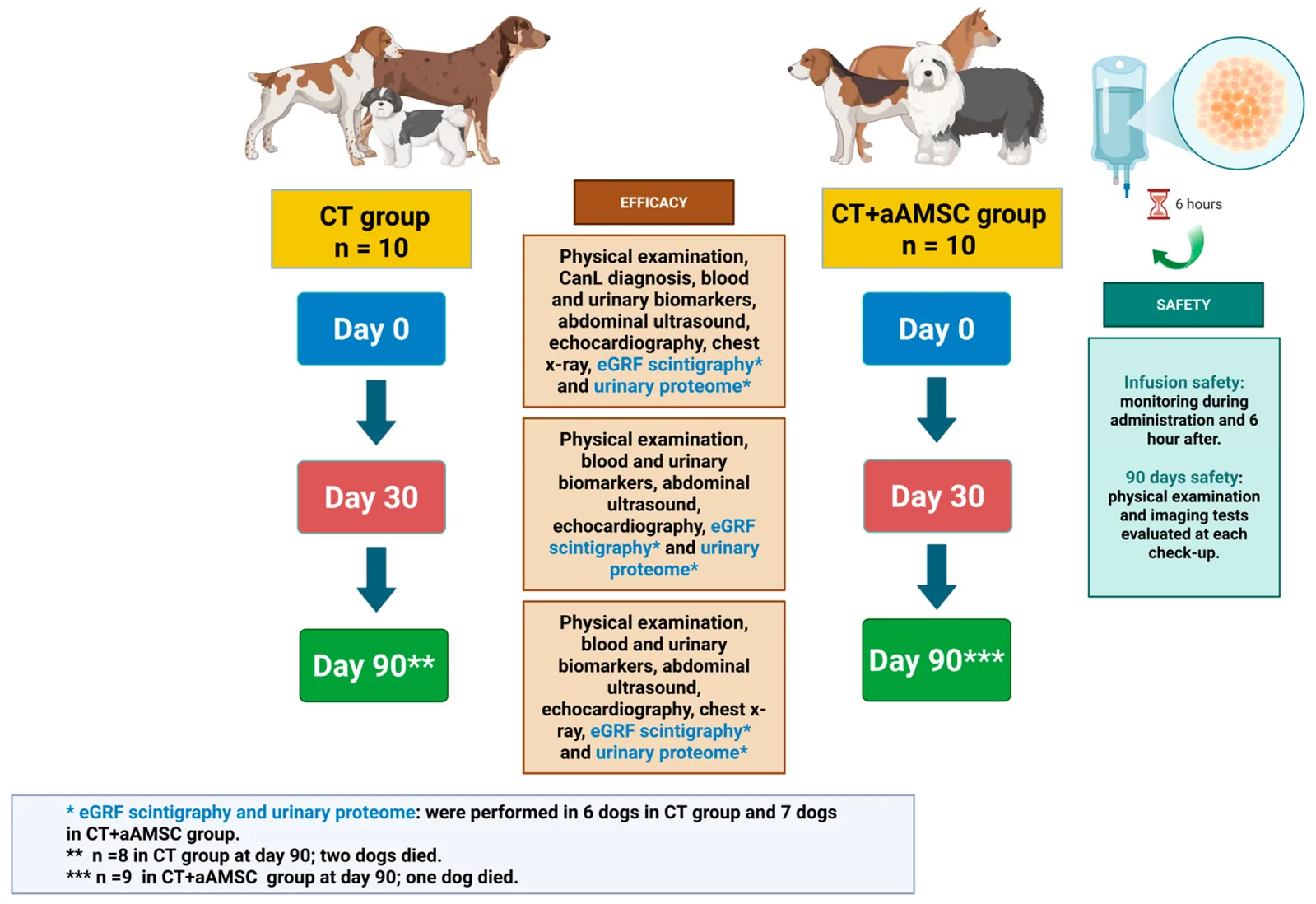
Flowchart showing the study groups (CT and CT + aAMSCs groups), and the controls performed throughout the research period (0, 30 and 90 days), as well as the diagnostic tests for the evaluation and tolerability of cell therapy. Created in BioRender. González, M. (2025) https://BioRender.com/v5gyl0q (accessed on 19 April 2025).

**Figure 2:**
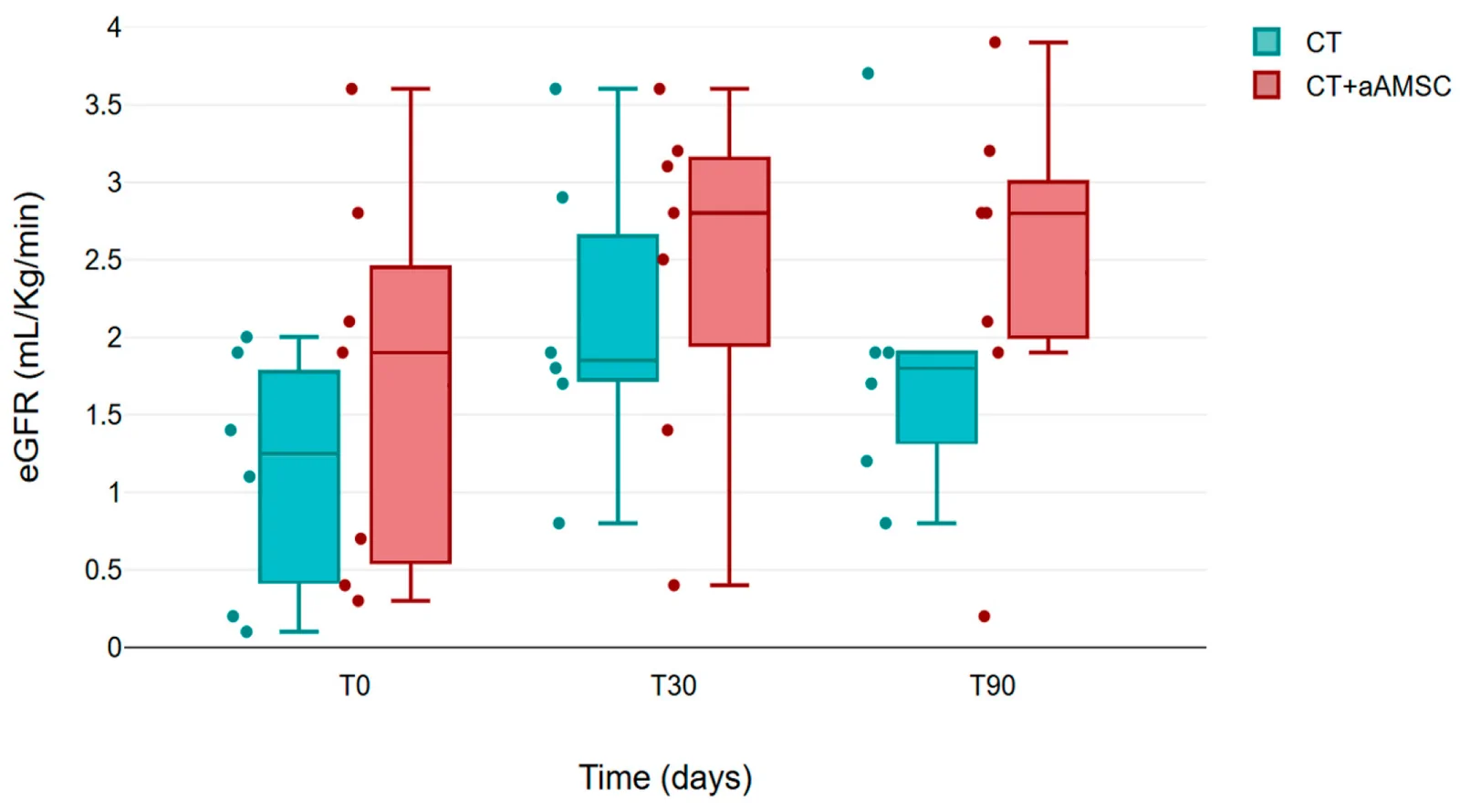
Estimated glomerular filtration rate (eGFR) at T0, T30, and T90 in the CT (blue) and CT + aAMSCs (red) groups. Individual measurements are overlaid on box plots. Horizontal lines indicate the medians, and boxes represent the 25th–75th percentiles.

**Figure 3:**
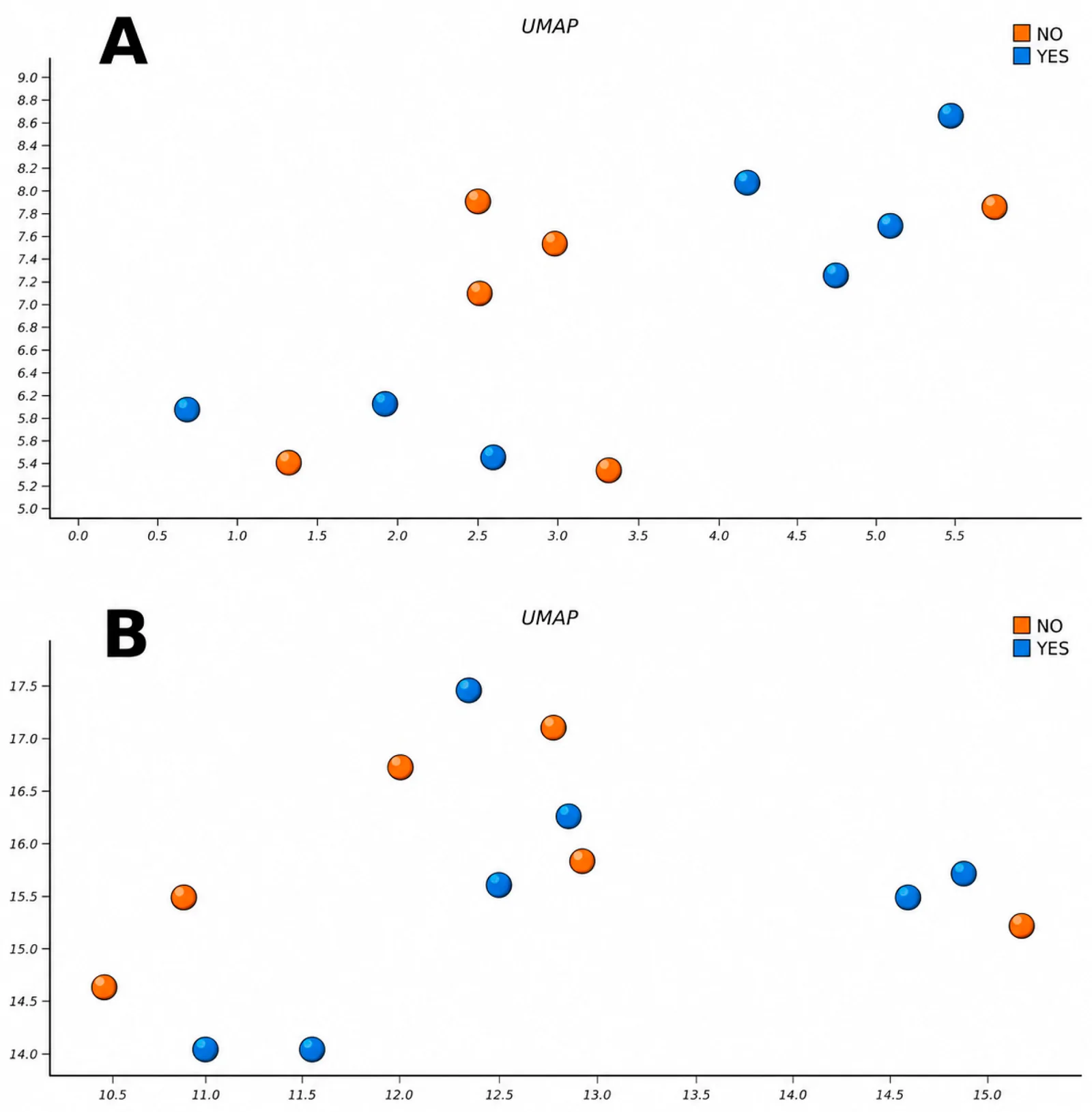
UMAP plots of urinary proteomic profiles at T30 (**A**) and T90 (**B**) in the CT (*n* = 6; orange) and CT + aAMSCs (*n* = 7; blue) groups. Each point represents one dog. “NO” denotes the CT group, whereas “YES” denotes the CT + aAMSCs group.

**Figure 4:**
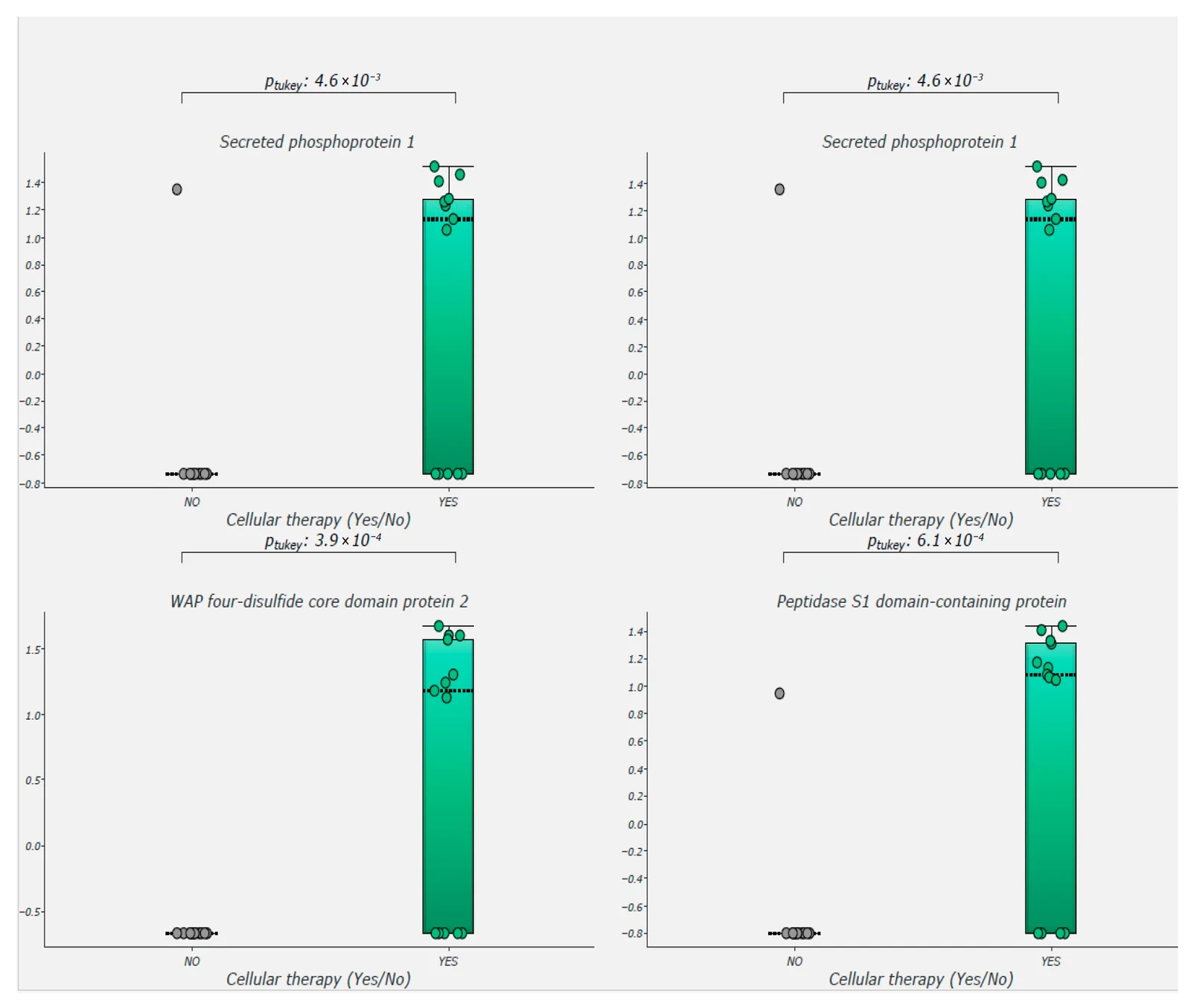
The relative abundance of the selected protein entries was compared between treatment groups. First, these values were normalized and Log2 transformed (Y axis). All four protein entries satisfied the filtering criteria (FC ≥ 2.85, *p* ≤ 0.007, and q < 0.07). The q-value represents the applied selection threshold, not an identical individual value for each entry. Individual Tukey-adjusted *p*-values are displayed above the box plots. Box plot representation was used to show these differences between CT (grey) and CT + aAMSCs (green) at T90. These graphics were created by Qlucore Omics Explorer version 3.6 Lund Sweden (https://qlucore.com).

**Table 1:** Blood biomarker measurements at T0, T30, and T90 in the CT and CT + aAMSCs groups. Data are presented as mean ± SD, except for pCr, pUrea, pSDMA, and phosphorus, which are presented as median (25th–75th percentiles).

Blood Biomarker	Time (Days)	*n*	CT	*p*-Time	*n*	CT + aAMSCs	*p*-Time	*p*-Group
pCr **(mg/dL)**	T0	10	1.35 (0.68–2.15)	0.531	10	1.95 (0.78–3.33)	0.569	0.405
	T30	10	1.15 (0.78–1.50)		10	1.00 (0.80–2.45)		0.879
	T90	8	0.85 (0.73–1.38)		9	0.80 (0.70–1.85)		1.000
pUrea **(mg/dL)**	T0	10	70.50 (21.25–196.25)	0.569	10	66.50 (23.50–229.00)	0.569	0.762
	T30	10	85.00 (27.00–134.25)		10	46.50 (23.50–139.00)		0.623
	T90	8	42.50 (23.50–74.00)		9	27.00 (24.00–52.00)		0.499
pCysC **(mg/L)**	T0	10	0.30 ± 0.17	0.125	10	0.38 ± 0.16	0.081	0.342
	T30	10	0.24 ± 0.07		10	0.31 ± 0.18		0.621
	T90	8	0.17 ± 0.11		9	0.27 ± 0.13		0.102
pSDMA **(µg/dL)**	T0	10	20.00 (13.00–27.25)	0.112	10	19.5 (16.00–37.00)	0.197	0.880
	T30	10	20.00 (13.75–28.50)		10	18.50 (12.00–29.00)		0.596
	T90	8	15.00 (12.00–17.00)		9	13.00 (9.75–23.75)		0.791
Albumin **(g/dL)**	T0	10	2.02 ^a^ ± 0.32	0.009 *	10	2.22 ± 0.39	0.094	0.228
	T30	10	2.23 ± 0.50		10	2.48 ± 0.48		0.269
	T90	8	2.48 ^a^ ± 0.39		9	2.50 ± 0.37		0.926
Calcium **(mg/dL)**	T0	10	10.06 ± 0.70	0.416	10	10.49 ± 1.16	0.324	0.327
	T30	10	10.57 ± 1.41		10	11.08 ± 0.75		0.623
	T90	8	10.08 ± 0.97		9	10.86 ± 0.74		0.082
Phosphorus **(mg/dL)**	T0	10	4.80 (4.20–5.35)	0.206	10	5.35 (4.05–6.50)	0.767	0.677
	T30	10	4.80 (3.25–6.68)		10	4.50 (3.58–5.05)		0.910
	T90	8	4.35 (3.98–4.58)		9	4.10 (3.50–4.90)		0.773
Potassium **(mmol/L)**	T0	10	4.56 ± 0.41	0.392	10	4.31 ± 0.39	0.726	0.138
	T30	10	4.47 ± 0.91		10	4.17 ± 0.61		0.595
	T90	8	4.10 ± 0.28		9	4.23 ± 0.35		0.405

The *p*-Time columns show within-group comparisons across time points, whereas the *p*-Group column shows between-group comparisons at each time point. Values sharing the same superscript letter differ significantly (*p* < 0.05). An asterisk denotes statistical significance (*p* < 0.05).

**Table 2:** Urinary biomarker results of the CT and CT + aAMSCs groups at times 0, 30, and 90 days expressed by median (25th and 75th percentiles), except uTHP/c, which are depicted as mean ± SD.

Urinary Biomarker	Time (Days)	*n*	CT	*p*-Time	*n*	CT + aAMSCs	*p*-Time	*p*-Group
uNGAL/c **(ng/mg)**	T0	10	170.29 ^a^ (49.23–374.33)	0.047 *	10	121.21 ^b^ (26.01–221.42)	0.006 *	0.345
	T30	10	83.89 (34.82–234.31)		10	32.52 (5.48–92.84)		0.089
	T90	8	59.64 ^a^ (17.38–80.93)		9	4.61 ^b^ (2.13–89.51)		0.413
uTHP/c **(mcg/mg)**	T0	10	126.70 ± 165.05	0.882	10	146.81 ± 271.31	0.236	0.571
	T30	10	80.48 ± 94.15		10	43.51 ± 57.53		0.307
	T90	8	130.68 ± 252.29		9	27.48 ± 27.48		0.194
UPCR	T0	10	4.84 (3.53–7.47)	0.113	10	3.35 ^c^ (1.88–5.75)	0.019 *	0.212
	T30	10	4.67 (2.24–4.88)		10	2.29 (1.70–5.31)		0.211
	T90	8	2.09 (1.38–4.00)		9	1.28 ^c^ (0.85–3.47)		0.361

The *p*-Time columns show within-group comparisons across time points, whereas the *p*-Group column shows between-group comparisons at each time point. Values sharing the same superscript letter differ significantly (*p* < 0.05). An asterisk denotes statistical significance (*p* < 0.05).

## Data Availability

The raw data supporting the conclusions of this article will be made available by the authors on request.

## Supplementary Materials

The following supporting information can be downloaded at: https://www.mdpi.com/article/10.3390/ani16213483/s1, Table S1: Individual baseline characteristics, clinical staging, and longitudinal biochemical, urinary biomarker, and renal scintigraphy data of dogs with canine leishmaniosis receiving conventional treatment (CT) or combined with allogeneic adipose-derived mesenchymal stromal cells (CT + aAMSCs).

## Abbreviations

The following abbreviations are used in this manuscript:

aAMSCs	Allogeneic adipose-derived mesenchymal stem/stromal cells
AKI	Acute kidney injury
AMSCs	Adipose mesenchymal stem/stromal cells
bmMSCs	Bone marrow mesenchymal stem/stromal cells
CanL	Canine leishmaniosis
CKD	Chronic kidney disease
CT	Conventional treatment group
DDA	Data-dependent acquisition
DMSO	Dimethyl sulphoxide
eGFR	Estimated glomerular filtration rate
EMT	Epithelial-to-mesenchymal transformation
FBS	Fetal bovine serum
MSCs	Mesenchymal stem/stromal cells
PCA	Principal component analysis
pCr	Plasmatic creatinine
pCysC	Plasmatic cystatin C
pSDMA	Plasmatic symmetric dimethylarginine
pUrea	Plasmatic urea
TGF-β	Transforming growth factor-β
UMAP	Uniform Manifold Approximation and Projection
uNGAL	Urinary neutrophil gelatinase-associated lipocalin
UPCR	Urinary protein-to-creatinine ratio
uTHP	Urinary Tamm–Horsfall protein
